# Disproportionality Analysis of Oral Toxicities Associated with PI3K/AKT/mTOR Pathway Inhibitors Using the FAERS Database

**DOI:** 10.3390/ph18101580

**Published:** 2025-10-19

**Authors:** Monica Marni, Djamilla Simoens, Nicholas Romero, Walter Keith Jones, Simon Kaja

**Affiliations:** 1 Program in Pharmacovigilance, Stritch School of Medicine, Loyola University Chicago, 2160 S. First Ave., Maywood, IL 60153, USA; 2 Department of Molecular Pharmacology and Neuroscience, Stritch School of Medicine, Loyola University Chicago, 2160 S. First Ave., Maywood, IL 60153, USA; 3 Department of Ophthalmology, Stritch School of Medicine, Loyola University Chicago, 2160 S. First Ave., Maywood, IL 60153, USA

**Keywords:** stomatitis, alpelisib, capivasertib, everolimus, palbociclib, inavolisib, breast cancer, HR+, HER2−, PI3K/AKT/mTOR, CDK4/6, FAERS, pharmacovigilance

## Abstract

**Background**: Stomatitis is a common adverse event associated with targeted therapies for hormone receptor-positive, HER2-negative (HR+/HER2–) breast cancer, particularly those inhibiting the PI3K/AKT/mTOR pathway. While mTOR-inhibitor-associated stomatitis is well established, less is known about its occurrence with other kinase inhibitors in real-world settings. We performed a pharmacovigilance disproportionality analysis of the FDA Adverse Event Reporting System (FAERS) to evaluate stomatitis reports for alpelisib, capivasertib, everolimus, and palbociclib. **Methods**: Events were identified using four term sets—Stomatitis, Original Trial Terms (OTT), Comprehensive Trial Terms (CTT), and Stomatitis-Associated Main Terms (SAMT)—which reflect varying definitions and medical terminologies. Disproportionality analyses using reporting odds ratio (ROR), proportional reporting ratio (PRR), and Information Component (IC) were calculated with 95% confidence intervals. **Results**: All agents showed ROR and PRR >1, indicating higher odds and reporting proportions of stomatitis compared with other drugs. These findings were confirmed by IC analysis. Everolimus demonstrated the strongest association (ROR: 30.72 [29.61–31.88]), followed by alpelisib (ROR: 13.11 [11.79–14.58]) and palbociclib (ROR: 11.73 [11.35–12.11]). Capivasertib had the lowest reporting odds (ROR: 3.14 [1.81–5.43]), though limited by fewer reports. Differences between CTT and SAMT were minimal (~2%). **Conclusions**: These results support the use of the SAMT as an efficient screening tool. Furthermore, these findings underscore the need for optimized stomatitis detection and continued monitoring, particularly for PI3K and mTOR inhibitors, in both clinical trials and postmarketing surveillance.

## 1. Introduction

Breast cancer is one of the most common cancers worldwide. As the leading cause of cancer-related death in women under 50 years of age, substantial efforts have focused on increasing the understanding of breast cancer pathophysiology and developing novel treatments [1]. Despite reductions in breast cancer mortality, incidence rates continue to rise, in part due to more advanced and proactive screening practices and possibly increased prevalence of risk factors [2]. These risk factors can include obesity, physical inactivity, red meat consumption, alcohol use, smoking, and oral contraceptives [3].

Breast cancer subtypes are classified based on the presence or absence of hormone receptors (HR) and the human epidermal growth factor receptor 2 (HER2) protein, a tyrosine kinase oncogene. HR-positive breast cancers express either estrogen or progesterone receptors, enabling cancer cells to receive hormone signals that promote tumor growth [4]. Overexpression of HER2 results in excessive cancer cell proliferation and suppression of normal apoptotic signals; thus, HER2-positive cancers are generally more aggressive malignancies [4,5,6].

Typically, breast cancer is classified into four major subtypes: HR+/HER2+, HR+/HER2−, HR−/HER2+, and HR−/HER2−. Of these, HR+/HER2− is the most common, accounting for approximately 70% of diagnosed cases [4]. This subtype is generally associated with a favorable prognosis due to slower growth and responsiveness to hormone therapies [7]. In patients with advanced (Stage III) HR+/HER2− breast cancer, mutations frequently occur in the phosphoinositide-3-kinase (PI3K)/protein kinase B (AKT)/mammalian target of rapamycin (mTOR) pathway, accounting for up to 40% of mutations in this population [8]. Given the prevalence of such mutations, the PI3K/AKT/mTOR pathway has become an important therapeutic target [9].

The standard of care for HR+/HER2− breast cancer is a drug regimen consisting of endocrine therapy combined with a cyclin-dependent kinase 4/6 inhibitor (CDK4/6i) [6]. However, endocrine resistance often develops, resulting in a substantial loss of efficacy of endocrine-based therapies [4]. In October 2024, the FDA approved the PI3Kα inhibitor inavolisib (ITOVEBI™) in combination with the standard-of-care agents palbociclib and fulvestrant for adults with endocrine-resistant, PIK3CA-mutated, HR+/HER2−, locally advanced or metastatic breast cancer [10]. In the phase III INAVO120 trial (NCT04191499), stomatitis was reported as an adverse event in 51% of patients who received this treatment regimen [11]. Given its recent approval, real-world data for patients receiving inavolisib (ITOVEBI™) remain limited, and incidence rates of stomatitis should be carefully monitored as inavolisib (ITOVEBI™) progresses through two additional phase III trials, INAVO121 (NCT05646862) and INAVO122 (NCT05894239), and into the postmarketing phase [12,13].

Stomatitis is an umbrella term describing inflammation of the oral mucosal membranes, which may include the lining of the oral cavity, lips, gingiva, tongue, palate, and pharynx [14]. The condition is typically characterized by ulcers, blisters, erythema (redness), or desquamation (peeling) of these membranes [15,16]. Diagnosis usually relies on oral tissue appearance, occasionally supplemented with laboratory culture to exclude oral infection, and review of the medical history [15,16]. The etiology of stomatitis includes medications, radiation, viral or fungal infections, smoking, physical trauma, nutritional deficiencies, and autoimmune disorders [17]. To refine diagnosis, stomatitis is often subcategorized according to etiology, such as *Candida*-related stomatitis, herpetic gingivostomatitis, or autoimmune stomatitis [18].

The term “stomatitis” is usually used to describe the adverse effect induced by targeted agents, such as inavolisib. In contrast, oral mucosal inflammation caused by other chemotherapy agents is sometimes referred to as “oral mucositis” [19]. Notably, the term “stomatitis” does not appear in the most recent version (v5.0) of the Common Terminology Criteria for Adverse Events (CTCAE) from the U.S. Department of Health and Human Services; instead, the preferred term is “mucositis oral” [20]. At the same time, the *Encyclopedia of Otolaryngology*, *Head and Neck Surgery* lists stomatitis as synonymous with aphthous stomatitis and mucositis [21], while the Food and Drug Administration Adverse Event Reporting System (FAERS) database does not contain the term “mucositis”. Specifically, mucositis is defined as involving tissue thinning in addition to inflammation and is typically caused by chemotherapy and radiation; interestingly, no such comment on etiology is provided in the definition for stomatitis [17,22]. The lack of clear differentiation between stomatitis and oral mucositis, and the absence of consensus on which oral structures, such as the pharynx, are affected in stomatitis, contribute to the challenges of monitoring stomatitis incidence in clinical trials and during postmarketing pharmacovigilance.

While stomatitis is not generally a life-threatening condition, it can significantly diminish the quality of life of patients [15]. Risk for stomatitis-associated adverse events is included as label warnings for other chemotherapies targeting the PI3K/AKT/mTOR pathway, with mTOR-inhibitor-associated stomatitis (mIAS) being well documented in the literature [23].

The purpose of this pharmacovigilance disproportionality analysis is to provide a comprehensive analysis of the reporting rates of stomatitis associated with four HR+/HER2− breast cancer medications, alpelisib/Piqray^®^, capivasertib/Truqap™, everolimus/Afinitor^®^, and palbociclib/Ibrance^®^, using data from the FAERS database. In addition to a comparative analysis of stomatitis reporting rates between these drugs, this study evaluates the potential impact of different criteria and definitions of stomatitis as a safety signal.

Everolimus is an mTOR kinase inhibitor that received initial FDA approval in 2009 for the treatment of advanced renal cell carcinoma after failure of treatment with sunitinib or sorafenib, and for subependymal giant cell astrocytoma associated with tuberous sclerosis in patients requiring therapeutic intervention but not eligible for curative surgical resection. In 2012, it was approved for the treatment of advanced HR+/HER2− breast cancer in postmenopausal women, in combination with exemestane, after failure of treatment with either letrozole or anastrozole [24].

Palbociclib is a cyclin-dependent kinase (CDK) 4/6 inhibitor used alongside other medications in first-line therapy for HR+/HER2− advanced or metastatic breast cancers. The FDA initially granted accelerated approval for palbociclib in February 2015, in combination with letrozole, for the treatment of postmenopausal women with estrogen receptor (ER)-positive, HER2− advanced breast cancer as initial endocrine-based therapy for metastatic disease [25].

Alpelisib is a phosphoinositide 3-kinase alpha (PI3Kα) inhibitor approved in May 2019 by the FDA, in combination with fulvestrant, for postmenopausal women and men with HR+/HER2−, PIK3CA-mutated, advanced or metastatic breast cancer, as detected by an FDA-approved test, following progression on or after an endocrine-based regimen [26].

Capivasertib is an AKT inhibitor, also known as protein kinase B inhibitor, approved by the FDA in November 2023, in combination with fulvestrant, for adult patients with HR+/HER2− locally advanced or metastatic breast cancer with one or more PIK3CA/AKT1/PTEN alterations, as detected by an FDA-approved test, following progression on at least one endocrine-based regimen in the metastatic setting or recurrence on or within 12 months of completing adjuvant therapy [27].

**Figure 1:**
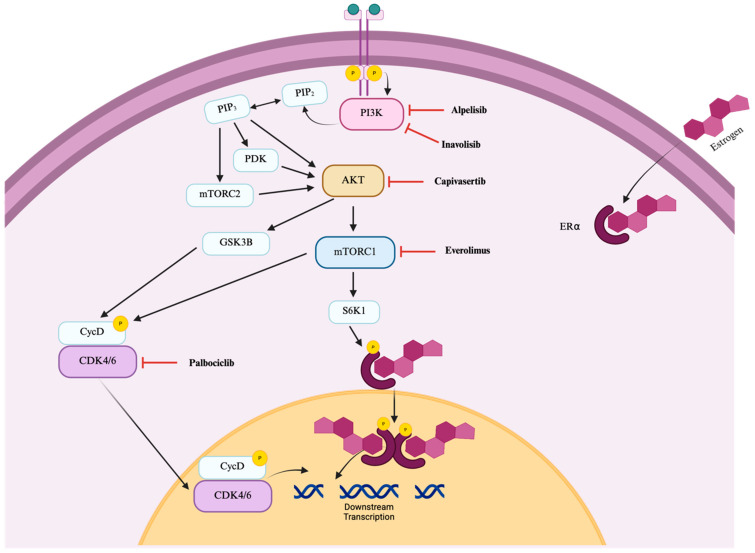
Molecular targets of protein kinase inhibitors for the treatment of HR+/HER2− breast cancer. Schematic representation of the PI3K/AKT/mTOR pathway. Alpelisib, capivasertib, and everolimus target PI3K, AKT, and mTORC1, respectively. In contrast, palbociclib inhibits CDK4/6 signaling downstream of mTORC1 activation. Abbreviations: PI3K: phosphatidylinositol-3 kinase, AKT: protein kinase B, mTORC1: mammalian target of rapamycin complex 1, mTORC2: mammalian target of rapamycin complex 2, PIP2: phosphatidylinositol 4,5-bisphosphate, PIP3: phosphatidylinositol 3,4,5-trisphosphate, PDK: phosphoinositide-dependent protein kinase, CycD: cyclin D, CDK4/6: cyclin-dependent kinase types 4 and 6, S6K1: ribosomal S6 kinase 1, Erα: estrogen receptor alpha, P: phosphorylated indication. Created in BioRender. Kaja, S. (2025) https://BioRender.com/q7my3m5.

Alpelisib, capivasertib, and everolimus disrupt the PI3K/AKT/mTOR pathway in a manner similar to inavolisib (Figure 1) and are associated with the incidence of stomatitis. Palbociclib, in contrast, acts further downstream (Figure 1) and was included in this analysis given its role in the inavolisib regimen as well as prior reports linking CDK4/6 inhibitors to an increased risk of stomatitis in breast cancer patients [28].

## 2. Results

**Figure 2:**
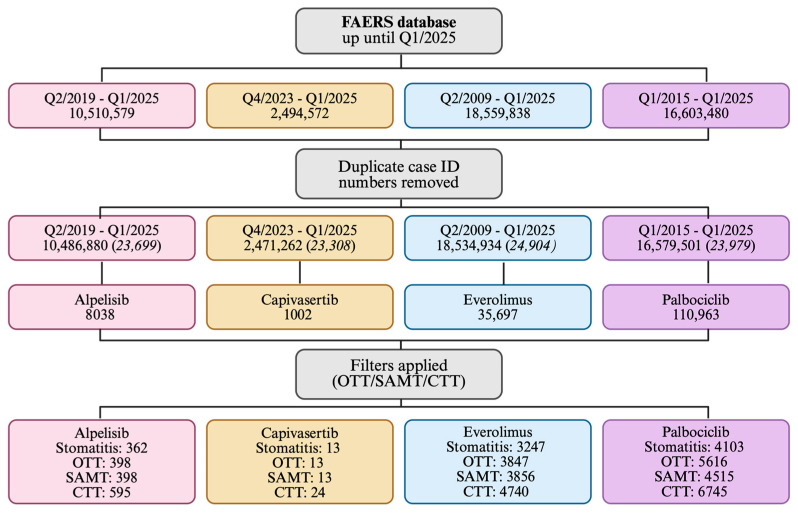
Flowchart of FAERS data extraction and filtering for stomatitis analysis. The FAERS database up to Q1 2025 was screened, with duplicate case IDs removed. Adverse event reports were retrieved for alpelisib, capivasertib, everolimus, and palbociclib. Stomatitis-associated cases were then identified using three predefined filters—Original Trial Terms (OTT), Stomatitis-Associated Main Terms (SAMT), and Comprehensive Trial Terms (CTT). The number of reports identified for each drug and filter is shown. Created in BioRender. Kaja, S. (2025) https://BioRender.com/pmx1mls.

Adverse event reports for alpelisib, capivasertib, everolimus, and palbociclib were extracted from the FAERS database, including data up to the end of Q1 2025 (31 March 2025). Initial extraction prior to duplicate removal yielded 8038 reports for alpelisib, 1002 for capivasertib, 35,697 for everolimus, and 110,963 for palbociclib (Figure 2).

**Figure 3:**
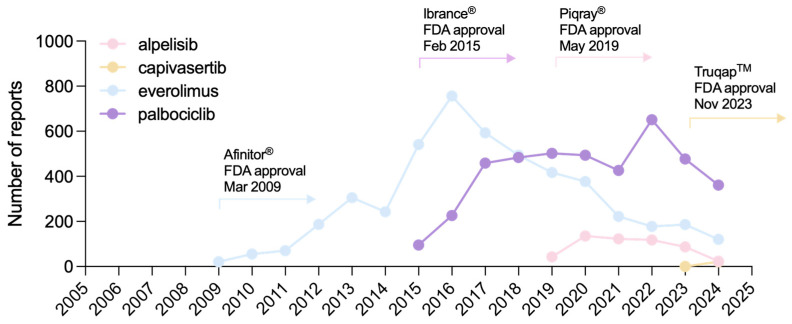
Graphical representation of the total number of reports submitted to the FAERS database for each drug since its FDA approval.

These differences in report numbers reflect the earlier FDA approvals of palbociclib (2015) and everolimus (2009) compared with the more recent approvals of alpelisib (2019) and capivasertib (2023) (Figure 3).

**Figure 4:**
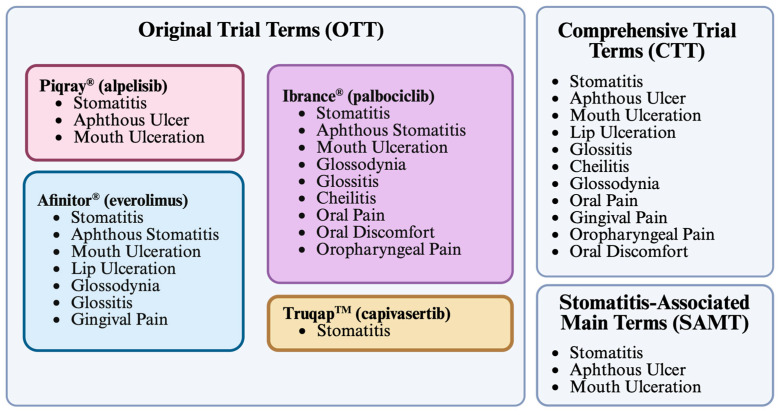
Description of term filters used to detect stomatitis-associated adverse events. The OTT, CTT, and SAMT filters were defined to screen for stomatitis-associated AEs for each drug. The OTT filter differed for each drug, as it was based on the specific terms used during the respective registration trial. Created in BioRender. Kaja, S. (2025) https://BioRender.com/alglb8u.

To identify stomatitis-related cases, three filter strategies were applied: Original Trial Terms (OTT), Comprehensive Trial Terms (CTT), and Stomatitis-Associated Main Terms (SAMT). The OTT filter was based on the event terms reported in the phase III trials of each drug and therefore differed across drugs. The CTT filter combined all terms used in the phase III trials of the four drugs. The SAMT filter focused on the three most common stomatitis terms reported in phase III trials: “stomatitis”, “aphthous ulcer”, and “mouth ulceration” (Figure 4). Some terms were excluded due to poor specificity or lack of compatibility with FAERS (e.g., “mucosal inflammation”, “aphthous stomatitis”).

Additional Medical Dictionary for Regulatory Activities (MedDRA) terms related to stomatitis and mucositis (e.g., “stomatitis haemorrhagic”, “stomatitis necrotizing”, “allergic stomatitis”, “mucositis management”, “immune-mediated mucositis”, “radiation mucositis”) were reviewed but not included in the final analysis. These terms were exceedingly rare, specifically three cases of “radiation mucositis” with everolimus, one case of “allergic stomatitis” with alpelisib, and eight cases—two “stomatitis haemorrhagic” and six “stomatitis necrotizing”—with palbociclib, and their inclusion would not have materially influenced the overall results.

Stomatitis-associated adverse events were detected for all four drugs. Using the CTT filter, 694 reports were identified for alpelisib, 24 for capivasertib, 5656 for everolimus, and 7927 for palbociclib. Corresponding counts for the SAMT filter were 408, 13, 4084, and 4695, respectively. For “stomatitis” alone, the numbers were 362, 13, 3247, and 4103, respectively (Table 1).

**Figure 5:**
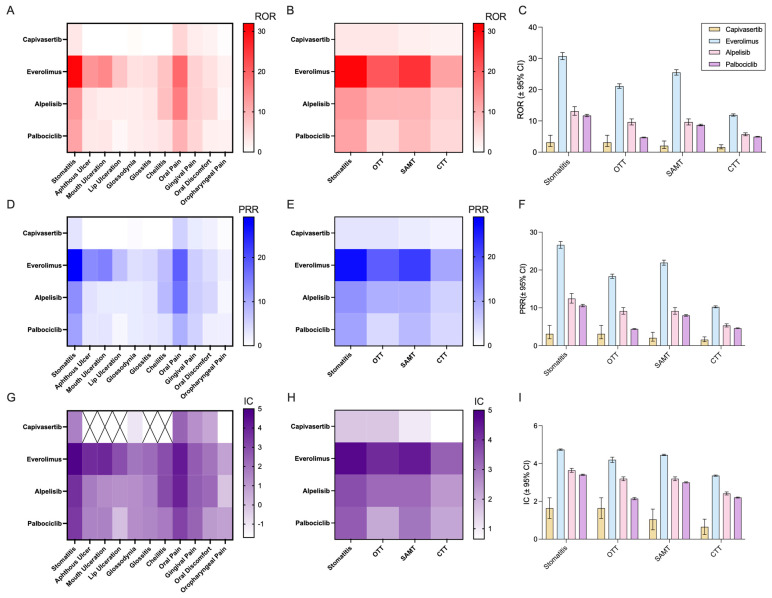
Disproportionality analysis of stomatitis-associated terms in FAERS: (**A**) Heatmap showing reporting odds ratios (ROR) for individual stomatitis-related terms across capivasertib, alpelisib, palbociclib, and everolimus. (**B**) Heatmap showing aggregated ROR values for stomatitis detected using the Original Trial Terms (OTT), Stomatitis-Associated Main Terms (SAMT), and Comprehensive Trial Terms (CTT) filters. (**C**) ROR values with 95% confidence intervals for the four different filters for capivasertib, alpelisib, palbociclib, and everolimus. (**D**) Heatmap showing proportional reporting ratios (PRRs) for individual stomatitis-related terms across capivasertib, alpelisib, palbociclib, and everolimus. (**E**) Heatmap showing aggregated PRR values for the different filters. (**F**) PRR values with 95% confidence intervals for the four different filters for capivasertib, alpelisib, palbociclib, and everolimus. (**G**) Heatmap showing the Information Component (IC) for individual stomatitis-related terms across capivasertib, alpelisib, palbociclib, and everolimus. (**H**) Heatmap showing aggregated IC values for all the different filters. (**I**) IC values with 95% confidence intervals for the four different filters for capivasertib, alpelisib, palbociclib, and everolimus.

Reporting odds ratios (RORs) and proportional reporting ratios (PRRs) were calculated for stomatitis events using “stomatitis” alone, or the three filters, SAMT, OTT, and CTT (Figure 5). All four drugs demonstrated ROR values greater than 1, indicating increased odds of stomatitis relative to other drugs in FAERS. PRR values were overall highly similar to ROR. Among the evaluated drugs, everolimus showed the highest disproportionality signal, while capivasertib showed the lowest. Alpelisib had a higher ROR than palbociclib, and the ROR for palbociclib was moderate in comparison.

**Figure 6:**
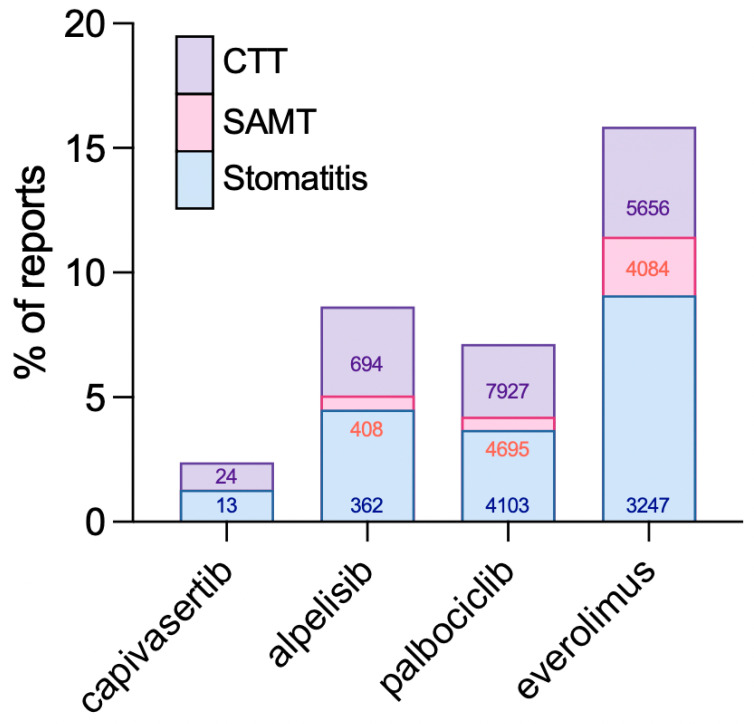
Percentage of stomatitis-associated reports identified for each drug in FAERS. The percentage of reports for capivasertib, alpelisib, palbociclib, and everolimus associated with stomatitis is shown. Cases were categorized according to predefined filters: Stomatitis-Associated Main Terms (SAMT), Comprehensive Trial Terms (CTT), or the single term “stomatitis”. Absolute case counts for each filter are indicated within the bars.

To evaluate the performance of the different filters, stomatitis cases captured by SAMT, CTT, and “stomatitis” alone were compared (Figure 6). The number of identified reports increased with broader term sets, as expected. The difference between SAMT and CTT was modest (~2% for most drugs), while differences between SAMT and “stomatitis” alone were minimal (0–1.5%). Differences between CTT and “stomatitis” ranged from 1% to 3.5%.

Across all filter strategies, everolimus consistently showed the highest proportion of stomatitis-related reports relative to its total FAERS cases. Alpelisib also showed a higher percentage of stomatitis-related reports compared with palbociclib.

## 3. Discussion

### 3.1. Mechanistic Considerations

We identified significant disproportionality of reporting for stomatitis across all four drugs, with everolimus showing the highest reporting odds ratio, alpelisib demonstrating intermediate values, palbociclib showing moderate reporting odds, and capivasertib displaying the lowest. These differences suggest that the reporting odds of stomatitis may vary depending on the molecular target within the PI3K/AKT/mTOR signaling pathway.

Everolimus, an mTOR inhibitor, produced the strongest signal, consistent with the well-documented phenomenon of mTOR-inhibitor-associated stomatitis (mIAS). mTOR plays a critical role in epithelial cell growth, mucosal turnover, and repair. Generally, its inhibition is thought to impair mucosal regeneration, thus leading to aphthous-like ulcerations [23].

Alpelisib, a PI3Kα inhibitor, also showed increased reporting odds compared with palbociclib. PI3K signaling influences both epithelial cell survival and immune regulation, and its inhibition may directly contribute to stomatitis pathophysiology. Palbociclib, in contrast, acts further downstream at CDK4/6 and demonstrated only a moderate signal, suggesting that inhibition of cell-cycle progression alone may not be sufficient to trigger the same degree of oral mucosal injury [9].

Capivasertib, an AKT inhibitor, showed the lowest reporting odds. While this might suggest that AKT inhibition is less directly involved in the pathways leading to oral inflammation, limited real-world data and the associated small number of reports due to the recent approval of the drug may skew results. Furthermore, as stomatitis was addressed during clinical development and on the drug label at the time of approval, this may reduce postmarketing reporting. Therefore, both limited real-world exposure and expectedness of the event could underestimate the true signal.

Taken together, these findings are consistent with a potential mechanistic gradient, where inhibition of PI3K or mTOR more strongly predisposes to stomatitis than targeting at the level of CDK4/6 or AKT. However, additional post-marketing data, especially for capivasertib, are necessary to confirm whether lower reporting odds translate to lower risk and/or reflect true biology or limitations of the current pharmacovigilance dataset.

### 3.2. Current State of Oral Toxicities and Cancer Treatments

Targeted and combination therapies, including the protein kinase inhibitors described here, have provided new and promising avenues for effective cancer treatment. However, toxicities affecting the oral cavity, such as stomatitis, continue to occur with these agents, albeit with different clinical presentations compared with toxicities caused by conventional chemotherapy or radiotherapy [29,30]. Although typically mild, these oral toxicities can substantially impair quality of life, causing pain and interfering with eating, drinking, and speaking [15]. Secondary complications, including dehydration and malnutrition, may arise and can further exacerbate stomatitis, sometimes leading to non-adherence or discontinuation of therapy. Medical attention and proactive evaluation of management strategies are, therefore, essential for patients receiving these drugs.

Baseline oral health examinations can help detect stomatitis early and identify patients with pre-existing conditions that may increase susceptibility to oral toxicities. Incorporating comprehensive oral health assessments into the clinical evaluation of drug candidates during phase trials may also facilitate the development of monitoring protocols that can be carried forward into the postmarketing setting.

Given the clinical significance of these toxicities, our analysis of stomatitis signals in FAERS provides important insight into how oral adverse events are reported with PI3K, AKT, mTOR, and CDK4/6 inhibitors, and highlights the need for systematic monitoring of oral health in patients treated with targeted therapies.

### 3.3. Recommendations for Identification of Stomatitis Safety Signals

The percentage of stomatitis cases captured by the SAMT filter differed slightly from the CTT filter, with an average difference of approximately 2%. While this difference may be relevant, it does not appear substantial enough to justify using all 11 terms included in the CTT filter over the three terms in SAMT. Differences between SAMT and “stomatitis” alone were even smaller (0–1.5%). However, a notable discrepancy was observed between “stomatitis” alone and the broader SAMT or CTT filters, suggesting that relying only on the term “stomatitis” would miss relevant cases that align with stomatitis events. To minimize the risk of underreporting, we recommend the use of the SAMT filter when screening for stomatitis safety signals in both clinical trials and postmarketing settings.

The SAMT filter captures pertinent stomatitis reports without being overly expansive, unlike the CTT filter. Although some cases may still be missed, the SAMT filter offers a practical balance that maximizes signal detection by relying on the most clinically relevant medical terms associated with stomatitis. Importantly, the use of SAMT can help prevent unintentional disregard of stomatitis, which is often underestimated as an adverse event in oncology, where patients often tolerate a high burden of side effects [31].

Our data may suggest that drugs targeting the PI3K/AKT/mTOR pathway, particularly PI3K and mTOR inhibitors, are associated with a higher risk of stomatitis than downstream targets such as CDK4/6. This finding is relevant in the context of combination regimens, where multiple protein kinase inhibitors may be administered simultaneously.

Awareness of the risk between stomatitis and targeted cancer therapies may help clinicians anticipate, monitor, and manage stomatitis more effectively. For example, providers may preemptively counsel patients about oral toxicities if their regimen includes both a PI3K and a CDK4/6 inhibitor, such as the FDA-approved combination of inavolisib and palbociclib. Increased awareness may also lead to more frequent reporting of stomatitis events, even if the true incidence remains unchanged. It should also be recognized that the number of reports varies significantly across drugs. Palbociclib, a first-in-class and first-line treatment for postmenopausal, metastatic HR+/HER2− breast cancer, regardless of mutational status, had the highest number of reports. In contrast, capivasertib had very few reports, most likely due to its recent market entry in 2023. As capivasertib use expands, its safety profile, including stomatitis risk, may become clearer with additional data.

Finally, infectious causes of stomatitis, including herpes simplex virus type 1 (HSV-1), herpes zoster, and *Candida albicans*, are common in oncology patients, particularly in the setting of chemotherapy or prolonged corticosteroid exposure [15,16]. As it is not possible to reliably distinguish infection-associated stomatitis from drug-related stomatitis in FAERS, reports of herpes or *Candida* infections were not excluded from the analysis.

### 3.4. Stomatitis and Oral Mucositis Treatment and the Need for Prophylaxis

There is no standardized treatment for stomatitis or oral mucositis. Common recommendations for patients with stomatitis include maintaining proper oral hygiene and avoiding products that could irritate the oral mucosa, such as spicy, acidic, or crunchy foods, alcohol, peroxide-based mouthwashes, and mint-flavored toothpaste [14]. Pain management strategies for stomatitis caused by targeted therapies or oral mucositis caused by chemotherapy/radiotherapy have included the use of ice chips, topical lidocaine, steroid ointments, low-level laser therapy, and, in severe cases, systemic analgesics [29,32,33].

Although not yet evaluated for stomatitis caused by targeted therapies, palifermin remains a potential option for future management of oral mucosal tissue damage. Palifermin, a recombinant human keratinocyte growth factor, was FDA-approved in 2004 to reduce the incidence and duration of oral mucositis in patients receiving intensive chemotherapy and radiotherapy for hematologic cancers [34]. The binding of keratinocyte growth factor to its receptor, some of which are expressed in the oral cavity, promotes epithelial proliferation, differentiation, and migration. Future investigations may evaluate palifermin for targeted therapy-associated stomatitis; however, caution is warranted, as chemotherapy/radiotherapy-induced oral mucositis is better understood than the pathobiology of stomatitis associated with PI3K/mTor/AKT pathway inhibitors [35,36]. In addition, some targeted therapies may produce distinct stomatitis phenotypes, such as mIAS, which more closely resembles recurrent aphthous stomatitis [23]. Even if palifermin is effective, its benefit may depend on the specific targeted agent involved.

In recent years, corticosteroid-containing mouthwashes have been studied for the prevention or mitigation of stomatitis and oral mucositis. These trials demonstrated particular efficacy of dexamethasone-based mouthwashes in breast cancer patients treated with chemotherapy, in breast cancer patients receiving everolimus (SWISH trial), and in head and neck cancer patients undergoing chemoradiation [37,38,39]. Some targeted therapies, such as inavolisib and everolimus, already mention corticosteroid-containing mouthwashes in their FDA labeling as an option for treatment or prevention of stomatitis. Expanding such labeling to other protein kinase inhibitors with known stomatitis risk may improve both provider and patient awareness and facilitate more consistent care [40]. This is particularly important given that pharmacies often dispense individualized “magic mouthwash” formulations that vary considerably in content [41]. Directly specifying dexamethasone-based mouthwashes on labels, with standardized dosing and swishing instructions, could help optimize symptom management. This is especially relevant since some “magic mouthwash” formulations contain antifungal agents such as nystatin, whereas the FDA label for everolimus specifically advises against antifungal use unless fungal infection is confirmed [42].

Although this study focused on stomatitis reports for four PI3K/mTOR/AKT pathway inhibitors individually, many targeted therapies are used in combination with chemotherapy or radiotherapy [9]. For patients receiving targeted therapies with even moderate stomatitis risk, preventive care and prophylaxis may be valuable in minimizing incidence and severity. This is especially relevant in regimens combining multiple stomatitis-associated agents, such as the FDA-approved combination of inavolisib with fulvestrant and palbociclib. Since both inavolisib and palbociclib have been linked to increased risk of stomatitis, proactive measures could substantially improve tolerability. Notably, while the FDA label for inavolisib recommends corticosteroid-containing mouthwash only after stomatitis develops, the everolimus label advises initiating dexamethasone alcohol-free mouthwash at the start of treatment. Amending labels to emphasize preventive strategies and specify effective treatments such as dexamethasone-based mouthwashes may ultimately improve patient outcomes. Previous studies have also highlighted the benefit of prophylaxis. For example, a study investigating mucositis associated with bone marrow transplant found that poor oral health at the time of transplant increased the risk of developing mucositis [43]. Prophylactic professional oral health care significantly reduced the incidence and severity of chemotherapy-induced oral mucositis, supporting its role as an effective preventive strategy in breast cancer patients [44]. Therefore, counseling patients on the importance of oral health combined with preventative measures such as mouthwashes may help decrease the impact of stomatitis-associated events.

### 3.5. Limitations of the Study

#### 3.5.1. Filters and Terms

The CTT filter used in this study comprises the terms applied in registration trials to detect stomatitis. However, it does not represent a comprehensive list of all possible terms that may signal a stomatitis event. Additional terms present in the FAERS database include *lip blister*, *mouth swelling*, *oedema mouth*, *oral blood blister*, *oral mucosal blistering*, *tongue ulceration*, and *tongue blistering*. Furthermore, the CTT filter in this analysis was restricted to the terms reported in registration trials and did not incorporate terms such as *mouth sores* or *mouth ulcers*—descriptors that often appear in FDA approval labels, prescribing information, or patient-facing literature. As stomatitis functions as an umbrella term encompassing oral mucosal inflammation, swelling, and ulceration, identifying discrete terms suitable for signal detection remains challenging. Further complexity arises in determining the anatomical boundaries of stomatitis, particularly when considering involvement of the pharynx. For example, it is not always clear whether pharyngeal ulcerations should be classified under stomatitis.

#### 3.5.2. Polypharmacy and Underlying Patient Conditions

The FAERS database contains limited clinical information, thus limiting our ability to adjust for confounders. Many of the drugs examined in this study are prescribed as part of complex regimens or treatment plans, which complicates attribution of adverse events to a single agent. Polypharmacy, as well as drug–drug and drug–food interactions, may further exacerbate certain adverse events or obscure their true cause [45], and polypharmacy in cancer patients is associated with oral mucosal changes. For example, patients taking four or more medications have an increased likelihood of developing xerostomia and present with an altered oral microbiome, which can lead to periodontal disease, caries, and oral mucositis even without a specific anticancer agent [46].

Underlying conditions or lifestyle choices can also predispose individuals to adverse events or even be the primary cause. For example, a patient who smokes and frequently consumes sour or spicy foods may develop a mouth sore independent of drug exposure. However, if that patient is taking a medication with mouth sores listed as a potential adverse effect, the event may still be reported as drug-related.

These background influences can also alter the severity of a stomatitis event, potentially amplifying its presentation compared to what might occur if the medication were taken in isolation, without external risk factors.

These factors may increase the background rate of stomatitis in FAERS reports and could inflate reporting odds ratios for individual drugs. Oral mucositis lesions compromise mucosal barriers and cause major infections, which may be reported and identified mistakenly as drug-induced toxicity rather than an oral cavity infection. As the FAERS database lacks detailed data on medication counts, oral hygiene, and infection status, it is impossible to distinguish these baseline risks from treatment-related events.

#### 3.5.3. Medical Terminology

In the literature, there is no standardized distinction between oral mucositis and stomatitis. In some contexts, the terms are used interchangeably, while in others, oral mucositis is defined more narrowly as oral mucosal inflammation secondary to chemotherapy or radiotherapy. This inconsistency is reflected in the Common Terminology Criteria for Adverse Events (CTCAE), which does not include “stomatitis” as a term but instead lists “mucositis oral”, defined as “a disorder characterized by ulceration or inflammation of the oral mucosa”.

Although the FAERS database contains several additional or more specific terms such as “*stomatitis haemorrhagic*”, “*stomatitis necrotizing*”, “*allergic stomatitis*”, “*mucositis management*”, “*immune-mediated mucositis*”, and “*radiation mucositis*”, we verified that these were exceedingly rare. The use of clinical trial-based terminology allowed for alignment with established efficacy and safety datasets, and highlights the broader challenge of consistent adverse-event capture in pharmacovigilance databases related to the hierarchical structure of MedDRA terms.

The lack of clarity regarding terminology may contribute to incomplete capture or misclassification of stomatitis incidents in safety reviews.

While such distinctions may be relevant to clinical investigators and healthcare professionals, they are unlikely to resonate with patients. When reporting adverse events, patients often use colloquial terms such as “canker sores”, “mouth sores”, or describe symptoms like “burning in the mouth”, whereas databases rely on standardized medical terminology. For example, the SAMT filter—comprising stomatitis, aphthous ulcer, and mouth ulceration—may be more biased toward terminology familiar to clinicians and underrepresent patient-reported language. In practice, “mouth ulceration” is likely closer to the phrasing patients would use, whereas “stomatitis” or “aphthous ulcer” are more specialized terms. This discrepancy raises concerns that signals based on patient or consumer reports may be underdetected compared with those generated by healthcare professionals or pharmaceutical companies.

#### 3.5.4. Constraints of the FAERS Database

Inherently, the FAERS database cannot be used to determine the true incidence of adverse events. Instead, FAERS data are most appropriately applied to generate disproportionality measures, such as the reporting odds ratio (ROR), which reflect relative reporting patterns rather than absolute risk. Therefore, it is important to note that FAERS signals indicate whether an event is reported more often with one drug than with others, but they do not establish how frequently the event occurs in the treated population. Here, we report ROR, PRR, and IC analysis results. ROR is generally the most statistically robust metric for FAERS data. Unlike incidence rates reported in clinical trials, ROR represents the relative likelihood of an adverse event being reported for one drug compared to others, rather than the absolute probability of the event occurring. The PRR reports the proportion of the specific adverse events, and as expected, was overall similar to the ROR for the four drugs and the different filters. The IC is a Bayesian disproportionality measure used to detect drug–adverse event associations, which is particularly well suited for small datasets as it applies a shrinkage adjustment toward the null hypothesis when data are sparse or infrequent.

Reporting frequencies in FAERS are influenced by external factors such as label warnings, provider or patient awareness, media coverage, regulatory emphasis, and differences in manufacturer pharmacovigilance systems. Reports may also reflect comorbidities, lifestyle factors, or the underlying disease rather than the medication itself. Thus, while FAERS is valuable for detecting safety signals and generating hypotheses, it must be interpreted as complementary to clinical trial data and cannot substitute for incidence data derived from rigorously monitored studies.

## 4. Materials and Methods

This pharmacovigilance disproportionality analysis used data from the FAERS database, which contained 30,179,725 reports as of 31 March 2025 (end of Q1 2025). Reports were retrieved for the following therapies: alpelisib (Q2 2019–Q1 2025), capivasertib (Q4 2023–Q1 2025), everolimus (Q2 2009–Q1 2025), and palbociclib (Q1 2015–Q1 2025) (Figure 2 and Figure 3).

To capture reports associated with stomatitis comprehensively, four filter strategies were applied. The Original Trial Terms (OTT) filter used adverse event terms reported in the phase III clinical trials of each drug, as listed in the FDA label (Figure 4). The Comprehensive Trial Terms (CTT) filter combined the definitions applied across all four drugs’ phase III clinical trials. The Stomatitis-Associated Main Terms (SAMT) filter focused on the three most frequent stomatitis-related terms: “stomatitis”, “aphthous ulcer”, and “mouth ulceration”. In addition, the term “stomatitis” alone was analyzed (Figure 4).

Some terms were considered but excluded. For example, “aphthous stomatitis” was not included in SAMT because it is not indexed within FAERS. Similarly, “mucosal inflammation”, which appeared in the PALOMA-2 trial for palbociclib, was omitted from OTT due to its poor specificity for oral localization. Of note, the PALOMA-3 trial employed the term “oropharyngeal discomfort” rather than the “oral discomfort” used in PALOMA-2.

The selection of stomatitis-related terms was purposefully limited to those reported in registration trials for each agent to maintain consistency with terminology used in pivotal studies and regulatory labeling.

Data were extracted from the FAERS database, deduplicated, and analyzed using a custom Python script generated with the help of ChatGPT-4 (https://github.com/DSimoens/FAERS_PHARMACOVIGILANCE_ANALYSIS, last accessed on 7 October 2025). Case reports were deduplicated by matching primary ID, normalized drug name, route of administration, and dosage (Figure 2). Both generic and brand names were included in the search. The alpelisib dataset also included some reports for VIJOICE^®^, alpelisib marketed and indicated for PIK3CA-related overgrowth spectrum. Individual report counts for each drug and search term are provided in Table 1.

Disproportionality analyses were performed following the removal of duplicate reports. For each adverse event group, a two-by-two contingency table was constructed to compare the frequency of reports involving the drug of interest with those involving all other drugs in FAERS. From these data, disproportionality metrics, including the reporting odds ratio (ROR), proportional reporting ratio (PRR), and Information Component (IC), were calculated with 95% confidence intervals. A flowchart showing the data analysis strategy is shown in Figure 4. Statistical significance was assessed using Fisher’s exact test as well as chi-square testing, with and without Yates’ continuity correction (Supplementary Materials). Statistical values are derived from the underlying 2 × 2 contingency tables, and as such are intrinsic to the data itself. If the 95% confidence intervals around the ROR or PRR exclude 1.0, the measure is statistically significant at α = 0.05. Similarly, the observed reporting for the IC is statistically significant when the lower bound is above zero.

This pharmacovigilance disproportionality analysis was performed consistent with the recommendations outlined in the READUS-PV recommendations [47].

## 5. Conclusions

The expansion of targeted therapies in precision oncology has led to a clearer understanding of their class-specific toxicity profiles. Stomatitis is a common adverse event that has been reported with certain targeted therapies, including drugs targeting the PI3K/AKT/mTOR pathway. Although common, stomatitis is a nonspecific term that often leads to confusion in both clinical practice and research. The inconsistent delineation from oral mucositis, alongside different possible etiologies such as diet, infection, and medication use, complicates reliable identification and tracking of drug-associated stomatitis events.

This study addressed two primary objectives. First, we evaluated the impact of different screening approaches for identifying stomatitis events. We found that the SAMT filter provides a balanced method for detecting relevant reports by capturing meaningful stomatitis signals without overwhelming investigators with the broader, less specific terms included in the CTT filter. Second, we assessed stomatitis reporting odds across four drugs targeting the PI3K/AKT/mTOR pathway. Consistent with reports in the literature, everolimus demonstrated the strongest association with stomatitis, reflecting the well-recognized toxicity profile of mTOR inhibitors [23,39]. Importantly, we also observed a higher disproportional reporting rate associated with alpelisib compared to palbociclib, suggesting that PI3K inhibition may more directly contribute to stomatitis pathophysiology than CDK4/6 inhibition.

Despite the limitations of this study, our findings support the ongoing monitoring of stomatitis in HR+/HER2- breast cancer patients using protein kinase inhibitors. Further evidence is needed to fully understand the extent to which stomatitis occurs with AKT inhibitors and why stomatitis rates were substantially higher in the INAVO120 clinical trial for inavolisib.

## Figures and Tables

**Table 1:** Stomatitis-associated adverse event reports in FAERS by drug and search term.

Term	Capivasertib	Alpelisib	Palbociclib	Everolimus
**Total number of reports**	1002	8038	110,963	35,697
**Stomatitis**	13	362	4103	3247
**Aphthous Ulcer**	0	22	218	279
**Mouth Ulceration**	0	24	374	558
**Lip Ulceration**	0	1	6	14
**Glossodynia**	1	31	353	167
**Glossitis**	0	4	53	28
**Cheilitis**	0	16	103	74
**Oral Pain**	6	150	1242	775
**Gingival Pain**	1	17	215	79
**Oral Discomfort**	1	23	141	91
**Oropharyngeal Pain**	2	44	1119	344
**Total Reports: Stomatitis**	13	362	4103	3247
**Total Reports: SAMT**	13	398	4515	3856
**Total Reports: CTT**	24	595	6745	6745

## Data Availability

The original contributions presented in this study are included in the article/Supplementary Material. Further inquiries can be directed to the corresponding author. The Python code used for the analyses in this study and instructions for use are publicly available at GitHub at https://github.com/DSimoens/FAERS_PHARMACOVIGILANCE_ANALYSIS (last accessed on 7 October 2025). All data from the FDA Adverse Event Reporting System (FAERS) are publicly accessible through their respective portals.

## Supplementary Materials

The following supporting information can be downloaded at: https://www.mdpi.com/article/10.3390/ph18101580/s1. Table S1: Statistical parameters for alpelisib, capivasertib, everolimus, and palbociclib. Figure S1: Disproportionality metrics for individual adverse event terms for alpelisib, capivasertib, everolimus, and palbociclib.

## Abbreviations

The following abbreviations are used in this manuscript:

AKT	Protein kinase B (part of PI3K/AKT/mTOR pathway)
AKT1	AKT kinase isoform 1
CDK	Cyclin-dependent kinase
CDK4/6	Cyclin-dependent kinases 4 and 6
CTCAE	Common Terminology Criteria for Adverse Events
CTT	Comprehensive Trial Terms
ER	Estrogen receptor
FAERS	FDA Adverse Event Reporting System
FDA	U.S. Food and Drug Administration
HER2	Human epidermal growth factor receptor 2
HR	Hormone receptor
HSV	Herpes simplex virus
mTOR	Mammalian target of rapamycin
PDK	Phosphoinositide-dependent protein kinase
PI3K	Phosphoinositide-3-kinase
PIP2	Phosphatidylinositol 4,5-bisphosphate
PIP3	Phosphatidylinositol 3,4,5-trisphosphate
PRR	Proportional reporting ratio
PTEN	Phosphatase and tensin homolog
ROR	Reporting odds ratio
