# Supplementary material for: Disproportionality Analysis of Oral Toxicities Associated with PI3K/AKT/mTOR Pathway Inhibitors Using the FAERS Database
Source: Pharmaceuticals (Basel). 2025 Oct 19;18(10):1580. doi: 10.3390/ph18101580 (PMC12567227; doi:10.3390/ph18101580)

Supplementary Materials

Supplementary Table S1: Statistical parameters for alpelisib, capivasertib, everolimus, and palbociclib.

Drug(s)	Filter	Fisher's test	χ^2 test	χ^2 test with Yates correction
alpelisib	OTT	7.01×10^{-235}	$< 1 \times 10^{-308}$	$< 1 \times 10^{-308}$
alpelisib	Stomatitis	5.75×10^{-258}	$< 1 \times 10^{-308}$	$< 1 \times 10^{-308}$
alpelisib	SAMT	7.01×10^{-235}	$< 1 \times 10^{-308}$	$< 1 \times 10^{-308}$
alpelisib	CTT	5.34×10^{-232}	$< 1 \times 10^{-308}$	$< 1 \times 10^{-308}$
capivasertib	OTT	3.94×10^{-4}	1.52×10^{-5}	4.49×10^{-5}
capivasertib	Stomatitis	3.94×10^{-4}	1.52×10^{-5}	4.49×10^{-5}
capivasertib	SAMT	0.014	0.008	0.013
capivasertib	CCT	0.037	0.025	0.034
everolimus	OTT	$< 1 \times 10^{-308}$	$< 1 \times 10^{-308}$	$< 1 \times 10^{-308}$
everolimus	Stomatitis	$< 1 \times 10^{-308}$	$< 1 \times 10^{-308}$	$< 1 \times 10^{-308}$
everolimus	SAMT	$< 1 \times 10^{-308}$	$< 1 \times 10^{-308}$	$< 1 \times 10^{-308}$
everolimus	CTT	$< 1 \times 10^{-308}$	$< 1 \times 10^{-308}$	$< 1 \times 10^{-308}$
palbociclib	OTT	$< 1 \times 10^{-308}$	$< 1 \times 10^{-308}$	$< 1 \times 10^{-308}$
palbociclib	Stomatitis	$< 1 \times 10^{-308}$	$< 1 \times 10^{-308}$	$< 1 \times 10^{-308}$
palbociclib	SAMT	$< 1 \times 10^{-308}$	$< 1 \times 10^{-308}$	$< 1 \times 10^{-308}$
palbociclib	CTT	$< 1 \times 10^{-308}$	$< 1 \times 10^{-308}$	$< 1 \times 10^{-308}$

Supplementary Figure S1: Disproportionality metrics for individual adverse event terms for alpelisib, capivasertib, everolimus, and palbociclib.

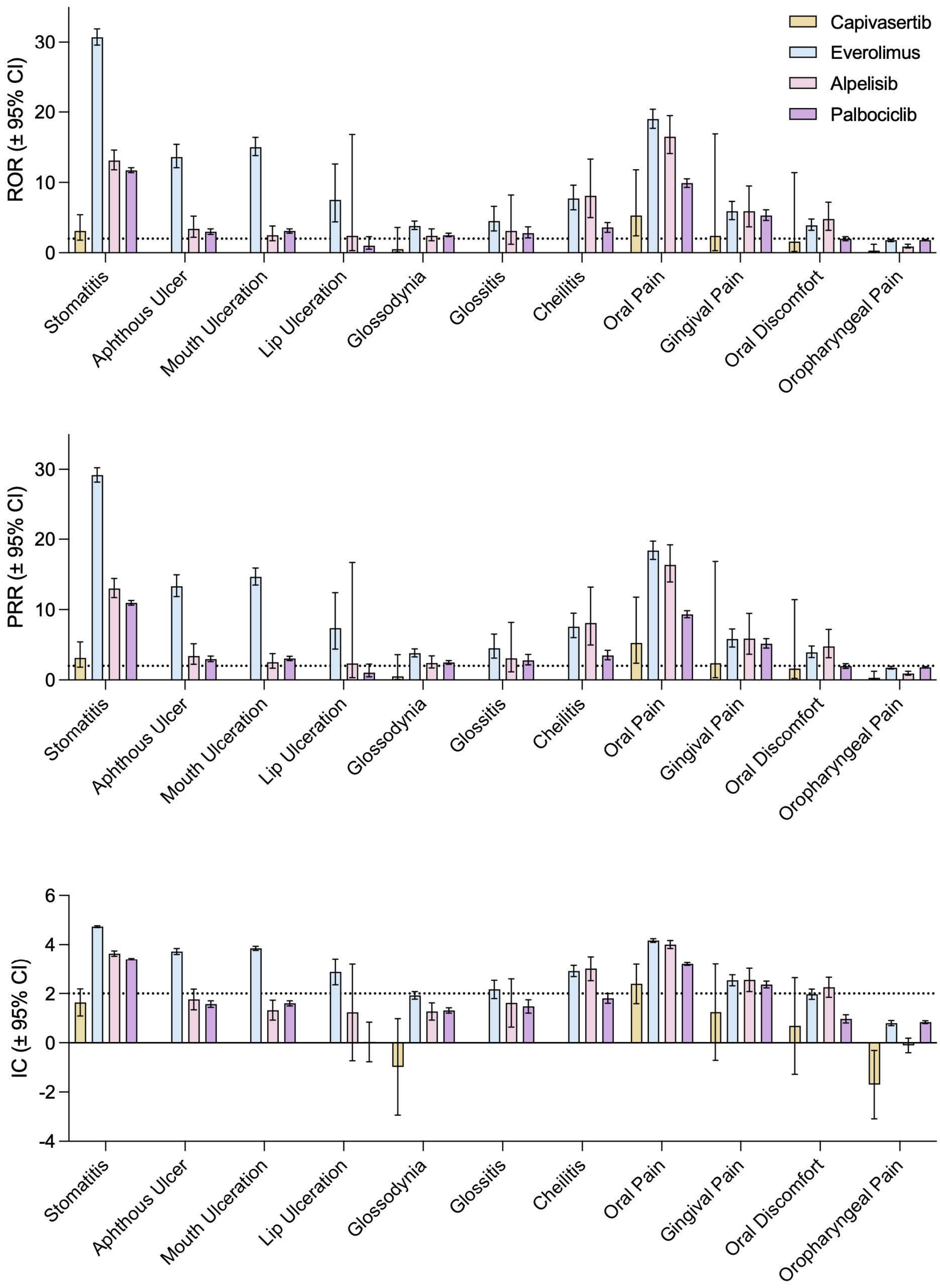

Supplement: Supplementary file 1 [file pharmaceuticals-18-01580-s001.zip › pharmaceuticals-3890056-supplementary.pdf]
